# Bullying behavior and mental health among college students in Sichuan, China: a cross-sectional study

**DOI:** 10.3389/fpubh.2026.1889019

**Published:** 2026-10-12

**Authors:** Yuanyuan Jing, Ruiqing Cao, Dajun Yang, Yang Liu, Juan Zhang, Honggang Hui, Yu Yao, Yuanyuan Deng, Yuanping Zhang, Xiong Ke, Keyuan Ran, Muhammad Najib Mohamad Alwi

**Affiliations:** 1 School of Management, North Sichuan Medical College, Nanchong, Sichuan, China; 2 Key Laboratory of Digital-Intelligent Disease Surveillance and Health Governance, North Sichuan Medical College, Nanchong, China; 3 School of Graduate Studies, Post Graduate Centre, Management and Science University, Shah Alam, Selangor, Malaysia; 4 School of Nursing, North Sichuan Medical College, Nanchong, Sichuan, China; 5 International Medical School, Management and Science University, Shah Alam, Malaysia; 6 Neuroscience and Mental Well-Being Centre (NeuroMIND), Management and Science University, Shah Alam, Malaysia

**Keywords:** anxiety, bullying, college students, depression, PTSD

## Abstract

**Background:**

Bullying is a known risk factor for poor mental health in adolescents. Although bullying research has largely focused on adolescent populations, college students represent a distinct group warranting dedicated attention. They shoulder unique developmental tasks including academic competition and career preparation, and exhibit behavioral characteristics of frequent engagement with digital platforms. This study examined the prevalence of bullying and its associations with depression, anxiety, and post-traumatic stress disorder (PTSD).

**Methodology:**

A cross-sectional survey was conducted among 1,120 students from August 18 to September 10, 2025. We recruited participants from a single college through class-based online channels using convenience sampling. Standardized scales assessed bullying experiences, anxiety, depression, PTSD symptoms, and psychological resilience.

**Results:**

A total of 241(21.5%) students reported being bullied, and 101(9.0%) reported bullying others. Mann–Whitney U tests showed no significant effects of age and ethnicity on the four mental health indicators (all *p* > 0.05). Gender and family residence groups exhibited statistically significant differences in GAD-7 and PHQ-9 scores; additionally, family residence groups differed in CD-RISC-25 scores (*p* < 0.05). Regression analyses indicated that traditional bullying victimization and cyberbullying victimization were significant factors associated with higher anxiety, depression and PTSD scores (*β* range: 0.280–0.561, all *p* < 0.001). Traditional bullying victimization was also associated with lower psychological resilience (*β* = −0.134, *p* = 0.009). The sensitivity analysis reveals that the inverse association observed between traditional bullying and certain indicators may be driven by suppression effects.

**Conclusion:**

The findings highlight the need for campus-based mental health screening, early identification of bullying involvement, and targeted bullying prevention initiatives among university students. Specifically, given that cyberbullying victimization showed a stronger association with PTSD symptoms compared to traditional bullying, trauma-focused interventions should be prioritized for cyberbullying victims to alleviate PTSD symptoms. In contrast, traditional bullying victims may benefit from resilience enhancement programs.

## 1 Introduction

Bullying, which is defined as aggressive behavior involving a power imbalance that causes substantial harm whether through repeated, prolonged, or a single severe incident (1, 2), remains a prevalent issue within educational settings (3–7). It can manifest in traditional forms, such as physical, verbal or relational bullying, or in the form of cyberbullying via digital communication technologies. Beyond physical and psychological harm, bullying is also related to poor academic performance, strained peer relationships and potential self-harm (8, 9). Given the focus of the present study, the associations between bullying, including traditional and cyberbullying victimization, and symptoms of depression, anxiety, and PTSD warrant particular attention (10–14). There is ample evidence indicating that bullying behavior does not disappear with the transition between educational stages, rather, it may persist into higher education while changing in form: face-to-face bullying may continue, and the widespread use of social media and other digital platforms creates additional settings in which bullying can occur, particularly in the form of cyberbullying (1, 13, 15–23). In contrast, less is known about bullying patterns among college students. Bullying among college students exhibits distinct characteristics shaped by the unique developmental context of emerging adulthood. Unlike adolescents who typically face structured environments with adult supervision, college students encounter more complex social dynamics involving greater autonomy, peer competition, and intensive digital engagement. College bullying frequently manifests through cyberbullying facilitated by extensive online academic and social activities, and involves different relational patterns influenced by the diverse residential, cultural, and socioeconomic backgrounds of adult learners. Although bullying has been widely studied in adolescent populations, research focusing specifically on college students remains comparatively limited (24–26). College students are a distinct developmental group, transitioning from late adolescence to emerging adulthood. This period is characterized by ongoing identity formation, greater autonomy and evolving peer relationships (27, 28). Therefore, findings derived from adolescent populations may not be directly generalizable to college populations. Despite growing attention to bullying in higher education, important gaps remain. Previous studies have often focused primarily on prevalence or victimisation, and few studies have examined traditional and cyberbullying together, distinguished victimization from perpetration, and assessed their associations with symptoms of PTSD, anxiety, and depression, as well as psychological resilience simultaneously (26, 29). Evidence from universities in China is particularly limited. This leaves a clear need for a school-based study that considers both bullying form and role and examines their relationships with a broader range of psychological indicators.

Bullying has become a major public health concern among young people in China, and its prevalence demonstrates considerable regional variation (14, 22, 30). Evidence from previous surveys indicates that the prevalence of bullying victims among college students differs across colleges/universities and regions, with reported prevalence ranging from 8.09 to 39.2% (14, 22, 23, 30–32). Notably, a multi-university survey in Hefei city, Anhui province found that 59.7% of Chinese college students had experienced at least one form of bullying during their lifetime (33). Moreover, in recent years, cyberbullying has gradually emerged as a significant social concern especially in youth population (15, 17, 34). During the COVID-19 pandemic, with young adults’ online usage frequency rising markedly and extended periods spent in virtual social settings, the prevalence of cyberbullying has shown a sustained upward trend, and this pattern has persisted even after the pandemic subsided (14, 35). Among college students in China, the prevalence of both traditional bullying and cyberbullying has also gained increasing attention. A recent study reported that 27.4% experienced bullying in the past year, including 15.6% through traditional bullying and 15.2% through cyberbullying (14). Additionally, international research similarly reveals the widespread nature of cyberbullying among college students: Hinduja and Patchin (36) identified cyberbullying as a significant concern among youth and young adults, documenting substantial proportions of both victimization and perpetration, while Kowalski et al. (37) conducted a comprehensive meta-analysis showing victimization rates of 20–40% across diverse educational settings. Given that bullying may lead to severe mental health consequences, particularly its close association with mental disorders such as PTSD, it is imperative to conduct a specialized survey targeting university students in China. This will provide a comprehensive understanding of the prevalence of bullying and its relationship with mental health issues.

Stress appraisal theory was used to organize the study variables and interpretation of their associations, and the theory proposes that psychological responses to stressful experiences are related to how individuals evaluate the significance of an event and the resources available to manage it (38). Within this framework, psychological resilience serves as a core indicator for assessing the functional state of the psychological adaptation system, reflecting the extent of psychological impairment associated with bullying. Bullying was conceptualized as a form of interpersonal stress that can occur in either offline or online settings. Traditional bullying and cyberbullying were analyzed separately. Meanwhile, victimization and perpetration were treated as two distinct roles within the context of bullying involvement. Victimization was regarded as a potentially threatening interpersonal experience, whereas perpetration represented another form of involvement that may be associated with an individual’s psychological state. Symptoms of anxiety, depression, and post-traumatic stress disorder (PTSD) were considered indicators of psychological distress, while psychological resilience was viewed as an indicator of adaptive psychological resources (39). It should be noted that the theoretical framework primarily serves to guide the selection and organization of research variables, providing a foundation for analysing the statistical associations among them.

Previous research has established the association between bullying experiences and adverse mental health outcomes, with several mechanisms identified to explain these relationships. Moore and Woodcock (40) demonstrated through their systematic review and meta-analysis that bullying victimization is significantly associated with increased anxiety and depression symptoms, potentially through pathways involving heightened threat perception and impaired emotional regulation. Regarding PTSD, Keles et al. (41) found that bullying constitutes a traumatic stressor that can lead to PTSD symptoms through processes involving fear conditioning and maladaptive memory formation. For psychological resilience, İme and Çakır (39) identified that bullying experiences erode resilience through the depletion of psychological resources and disruption of adaptive coping processes. Jawwad et al. (42) further confirmed that resilience mediates the relationship between bullying and mental health outcomes among university students. While these mechanisms have been primarily documented in Western or adolescent populations, their applicability to Chinese college students—who face unique academic pressures and cultural contexts—remains underexplored. The present study builds on these frameworks to examine how traditional bullying, cyberbullying, and their respective victimization experiences are associated with depression, anxiety, PTSD symptoms, and psychological resilience among Chinese college students, with particular attention to the role of psychological resilience as both an outcome and potential mediator.

Accordingly, the present study aimed to: (1) investigate the prevalence of traditional and cyberbullying victimization and perpetration among college students at a university in Nanchong, Sichuan Province; (2) explore the associations between these forms of bullying and anxiety, depression, PTSD symptoms and psychological resilience; and (3) describe the observed patterns of association across different forms of bullying and roles. It was hypothesised that victimization would be associated with higher levels of anxiety, depression, and PTSD symptoms, as well as lower psychological resilience, and that these associations may differ depending on the bullying role. Due to the cross-sectional design, all findings were interpreted as statistical associations rather than causal relationships. And these findings may provide preliminary evidence to inform school-based mental health screening and the early identification of bullying and psychological distress, as well as targeted support.

## 2 Methodology

### 2.1 Participants

This study employed a convenience sampling strategy to recruit participants between August 18 and September 10, 2025. The sampling frame consisted of full-time undergraduate students enrolled at North Sichuan Medical College. An *a priori* power analysis was conducted using G*Power software. Following Cohen’s recommendations for effect size estimation in social science research, a medium effect size (f^2^ = 0.15) was assumed, representing a reasonable estimate for studies examining the associations between bullying involvement and mental health outcomes. With a significance level of α = 0.05 and statistical power set at 0.80, the required minimum sample size for multiple regression analyses involving up to 12 predictors was estimated to be approximately 112 participants. Therefore, the final sample of 1,120 participants provided more than adequate statistical power (exceeding 0.95) to detect meaningful associations across all mental health outcomes examined in this study.

The inclusion criteria were: (1) 18–29 years old, full-time college students; (2) able to communicate effectively in Chinese; (3) having reliable access to the internet through the survey period; (4) absence of severe illness at the time of survey, with the capacity to complete the questionnaire independently; (5) voluntary participation in the study and giving informed consent. All participants were informed of the research objectives, potential benefits, and possible risks before completing the questionnaire. It was explicitly stated that participation was entirely voluntary, and participants could choose to join or withdraw at any time during the study.

The exclusion criteria were: Questionnaires were excluded if they met any of the following conditions: (1) Incomplete responses or significant data gaps; (2) Logical inconsistencies in key items; (3) Incorrect responses to attention-check questions (trap questions); (4) Duplicate submissions from the same IP address.

### 2.2 Procedures

Prior to survey distribution, researchers first contacted counselors from various departments and faculties through offline communication to obtain their consent and support for the study. Upon receiving informed consent from the counselors, researchers generated a unique QR code for the questionnaire via the Credamo platform. Counselors then disseminated the survey by posting it in corresponding grade-level and major-specific WeChat group chats, inviting students to voluntarily participate in the survey. Students accessed and completed the questionnaire by scanning the QR code with their mobile devices. Upon survey completion, all data were automatically collected and stored in the Credamo platform, from which researchers later exported the dataset for further analysis. To minimise selection bias, the survey was disseminated across multiple departments and grade levels to ensure representation from diverse academic programs and student cohorts. Additionally, all questionnaire items were designated as mandatory, and attention check questions were embedded to identify and exclude inattentive or low-quality responses.

The questionnaire’s opening page provided a brief explanation of the research objectives and significance. Participants voluntarily completed the survey after reviewing this information. To enhance data quality and minimize invalid responses, multiple quality control measures were implemented during the survey process. These included restricting each IP address to a single submission to prevent duplicate responses. Additionally, trap questions were embedded within the questionnaire to identify responses lacking diligence or containing obvious logical errors. Furthermore, all questions were designated as mandatory to minimize missing data.

### 2.3 Data collection instruments

The questionnaire consisted of two sections. The first section collected demographic information, including gender, age, ethnicity, grade level, major, family residence, whether or not they were an only child, and parents’ level of educational. The second section contained the Chinese versions of standardized instruments that had been validated for use in assessing traditional bullying, cyberbullying, symptoms of anxiety, depression, and PTSD, as well as psychological resilience. The score ranges and interpretation criteria for each instrument are summarized in Table 1.

**Table 1:** Measurements of bullying experience and mental health problems.

Instrument	Score range	Severity classification/cut-off
PHQ-9 (Patient Health Questionnaire-9)	0–27	0–4: minimal or none; 5–9: mild; 10–14: moderate; 15–19: moderately severe; 20–27: severe. A score ≥ 10 indicates clinically significant depression.
GAD-7 (Generalized Anxiety Disorder-7)	0–21	0–4: minimal or none; 5–9: mild; 10–14: moderate; 15–21: severe. A score ≥ 10 indicates clinically significant anxiety.
PCL-5 (PTSD Checklist for DSM-5)	0–80	A cut-off of ≥ 33 suggests probable PTSD.
OBVQ (Olweus Bully/Victim Questionnaire)/CPS & CVS	Frequency-based	Experiencing school bullying with a frequency score ≥ 1 was classified as having been a victim/bully of school bullying. Similarly, reporting cyberbullying with a frequency ≥ 1 (frequency score > 9) was considered indicative of having perpetrated or experienced cyberbullying.
CD-RISC (Connor–Davidson Resilience Scale)	CD-RISC-25: 0–100;	No fixed clinical threshold. Higher scores reflect greater resilience.

#### 2.3.1 Bullying experience assessment scale

The Chinese version of the Olweus Bullying/victimization Questionnaire (OBVQ) was used to measure traditional bullying and victimization. The scale was originally developed by Olweus (43) and adapted for the Chinese context by Zhang and Wu (44). Peng et al. (45) used and validated it to ensure its applicability among contemporary Chinese students. Previous studies have also employed the OBVQ to assess bullying experiences among college students. For example, Zhao et al. (46) applied the OBVQ to examine past bullying experiences in Chinese university students. The studies demonstrate the widespread application of the OBVQ in college student populations. Cyberbullying was assessed using the Cyberbullying Perpetration Scale (CPS) and Cyberbullying Victimization Scale (CVS) for Chinese Adolescents. This instrument was originally developed by Tang et al. (47) and further validated by Liu (48), and it specifically captures digital harassment behaviors within the Chinese social media environment. The scale covers core dimensions, including cyberbullying perpetration and victimization. In the present study, the Cronbach’s α coefficients for the OBVQ and cyberbullying scale were 0.96 and 0.98, respectively, indicating excellent internal consistency reliability in our college student sample. Both instruments use a frequency-based scoring system, with scores of ≥1 on the corresponding OBVQ subscale or total scores of >9 on the CPS or CVS indicating the occurrence of the corresponding bullying perpetration or victimization experience. Participants were then categorized according to their role in bullying and its form, as either traditional bullying perpetrators, cyberbullying perpetrators, traditional bullying victims or cyberbullying victims. Those with a frequency score of at least 1 on either perpetration scale were defined as total perpetrators, while those with a frequency score of at least 1 on either victimization scale were defined as total victims.

#### 2.3.2 Mental health assessment scales

The Chinese version of the 7-item Generalized Anxiety Disorder scale (GAD-7) was used to assess anxiety symptoms (49), which is based on the original framework by Spitzer et al. (50), and determined the symptom frequency over the preceding 2 weeks. Depressive symptoms were measured using the Chinese version of the 9-item Patient Health Questionnaire (PHQ-9) (51), which was originally developed by Kroenke, Spitzer et al. (52) to evaluate symptom severity during the same period. PTSD symptoms were measured using the Chinese version of the PTSD Checklist for DSM-5 (PCL-5) (53), developed by Weathers (54), while psychological resilience was measured using the Chinese version of the 25-item Connor-Davidson Resilience Scale (CD-RISC-25) (55, 56), which was originally developed by Connor and Davidson (57). In the present study, the Cronbach’s α coefficients for the GAD-7, PHQ-9, PCL-5, and CD-RISC-25 were 0.94, 0.91, 0.98 and 0.99 respectively, indicating excellent internal consistency. These self-report instruments were used to assess the severity of anxiety, depressive, and PTSD symptoms and the level of psychological resilience; they were not used to establish clinical diagnoses.

### 2.4 Ethical considerations

This study received approval from the Academic Ethics Committee of North Sichuan Medical College (No. 2025020). All Participants were informed of the study purpose and procedures, the voluntary nature of participation, their rights, and any potential risks before the survey began. Participants were considered to have provided informed consent if they proceeded to complete and submit the questionnaire after reading this information. Confidentiality was maintained throughout the study, and data was processed anonymously.

### 2.5 Data analysis

Data was exported from the Credamo platform into IBM SPSS Statistics version 27.0. Continuous variables were presented as mean ± standard deviation (x̄ ± SD) or medians (interquartile ranges), according to their distributions. Categorical variables were presented as frequencies and percentages. Independent-samples t-tests or Mann–Whitney U tests were used for two-group comparisons, whereas one-way analysis of variance (ANOVA) or Kruskal-Wallis H tests were applied for comparisons among multiple groups. Differences in categorical variables were examined using Pearson’s chi-square test or Fisher’s exact test, as appropriate.

As demographic characteristics were mainly categorical variables and were analysed using the appropriate group comparison tests described above, Bivariate correlation analyses were conducted to examine the correlations among the four bullying variables and their correlations with the mental health outcomes. Correlations among the bullying variables were also inspected to characterize behavioral overlap and to identify potential collinearity before multivariable modelling. Separate univariable linear regression analyses were conducted for each outcome: GAD-7, PHQ-9, PCL-5, and CD-RISC-25 total scores. The independent variables included traditional bullying perpetration, traditional bullying victimization, cyberbullying perpetration and cyberbullying victimization, each of which was coded as a continuous variables. These were considered alongside demographic variables, which were coded as categorical variables.

Separate multivariable linear regression models were fitted for the PHQ-9, GAD-7, PCL-5, and CD-RISC-25 total scores. For each dependent variable, factors associated with the corresponding scale score at *p* < 0.05 in the univariable analyses were entered into the primary model. Sensitivity analyses were then conducted by entering all bullying-related and demographic variables simultaneously using the Enter method to assess the robustness of the observed associations. Model outputs included unstandardized coefficients, standard errors, standardized coefficients, t-values, *p*-values, and multicollinearity diagnostics (variance inflation factor, VIF). A *p*-value < 0.05 was considered statistically significant.

## 3 Results

A total of 1,314 questionnaires were collected via Credamo online questionnaire platform. Following data quality screening, 194 invalid questionnaires were excluded due to incomplete responses, logical inconsistencies in key items or failure to trap questions. Ultimately, 1,120 questionnaires were included in the statistical analysis, yielding an effective rate of 85.24%.

### 3.1 Participant characteristics

Demographic characteristics of the participants—including gender, age, ethnicity, hometown location, academic year, and major—showed that females, students aged 18–21, Han students, and students from rural areas accounted for larger proportions. Freshmen and students majoring in Preventive Medicine represented the largest grade and major groups, respectively (see Table 2). Descriptive statistics for bullying exposure and mental health indicators are presented in Table 3. Scores for traditional bullying victimization and perpetration were generally low. Anxiety, depression, and PTSD symptom scores were modest, while psychological resilience was at a moderate level.

**Table 2:** Demographic characteristics of participants (*n* = 1,120).

Variable	Category	Number (n)	Proportion (%)
Gender	Female	832	74.3
	Male	288	25.7
Age (years)	18–21	915	81.7
	>21	205	18.3
Ethnicity	Han	1,034	92.3
	Minority	86	7.7
Family residence	Rural	636	56.8
	Urban	484	43.2
Grade	Freshman	363	32.4
	Sophomore	221	19.7
	Junior	217	19.4
	Senior	298	26.6
	Fifth year	21	1.9
Major	Preventive Medicine	146	13.0
	Nursing	139	12.4
	Public Administration	113	10.1
	Financial Management	100	8.9
	Clinical Medicine	93	8.3
	Information Management and Information Systems	96	8.6
	Medical Imaging	84	7.5
	Midwifery	83	7.4
	Social Work	44	3.9
	Medical Laboratory Science	38	3.4
	Stomatology (Dentistry)	29	2.6
	Optometry (Medical)	29	2.6
	English	21	1.9
	Optometry	17	1.5
	Other	88	7.9

**Table 3:** Descriptive statistics of participants (*n* = 1,120).

Variable	*M* ± *SD*	*Mdn* (*P_25_*, *P_75_*)	Range
Traditional bullying victimization	0.60 ± 1.94	0.00 (0.00, 0.00)	0–16
Traditional bullying perpetration	0.28 ± 1.58	0.00 (0.00, 0.00)	0–17
Cyberbullying victimization	9.37 ± 2.26	9.00 (9.00, 9.00)	9–32
Cyberbullying perpetration	9.50 ± 2.27	9.00 (9.00, 9.00)	9–38
Anxiety	2.57 ± 3.66	1.00 (0.00, 4.00)	0–21
Depression	3.03 ± 4.21	1.00 (0.00, 5.00)	0–27
PTSD	4.24 ± 10.18	0.00 (0.00, 3.00)	0–80
Resilience	55.02 ± 26.24	57.00 (44.00, 72.75)	0–100

### 3.2 Mental health according to demographic characteristics

Mann–Whitney U tests (see Table 4) indicated statistically significant differences in GAD-7 and PHQ-9 scores across gender and family residence groups. Females reported significantly higher anxiety (*Mdn* = 1.00, *Z* = 2.722, *p* < 0.010) and depression (*Mdn* = 1.00, *Z* = 2.098, *p* = 0.036) scores than males. Similarly, students from rural areas had significantly higher anxiety (*Mdn* = 1.00, Z = −3.222, *p* < 0.010) and depression (*Mdn* = 2.00, *Z* = −2.175, *p* = 0.030) scores compared to urban students. In addition, students from rural areas had significantly lower resilience scores than those from urban areas (*Mdn* = 56.00, *Z* = 2.201, *p* = 0.028).

**Table 4:** Mental health indicators by demographic characteristics (*n* = 1,120).

Variable	N	Anxiety *Mdn* (*P_25_*, *P_75_*)	Depression *Mdn* (*P_25_*, *P_75_*)	PTSD *Mdn* (*P_25_*, *P_75_*)	Resilience *Mdn* (*P_25_*, *P_75_*)
Gender					
Male	288	0.00 (0.00, 3.00)	0.00 (0.00, 5.00)	0.00 (0.00, 2.00)	59.00 (34.25, 74.00)
Female	832	1.00 (0.00, 4.00)	1.00 (0.00, 5.00)	0.00 (0.00, 3.00)	57.00 (45.25, 72.00)
*Z*		2.722	2.098	1.651	0.508
*P*		<0.01	0.036	0.099	0.612
Ethnicity					
Han	1,034	1.00 (0.00, 4.00)	1.00 (0.00, 5.00)	0.00 (0.00, 3.00)	57.00 (44.00, 73.00)
Minority	86	1.00 (0.00, 4.00)	2.00 (0.00, 6.00)	0.00(0.00, 3.00)	58.00 (45.50, 72.25)
*Z*		−0.858	−1.284	−1.130	−0.282
*P*		0.391	0.199	0.259	0.778
Family residence					
Urban	484	0.00 (0.00, 4.00)	1.00 (0.00, 4.00)	0.00 (0.00, 2.00)	60.00 (46.00, 74.00)
Rural	636	1.00(0.00, 4.00)	2.00 (0.00, 5.00)	0.00 (0.00, 3.00)	56.00 (43.00, 72.00)
*Z*		−3.222	−2.175	−1.614	2.201
*P*		<0.01	0.030	0.107	0.028
Age					
18–21	915	1.00 (0.00, 4.00)	1.00 (0.00, 5.00)	0.00 (0.00, 3.00)	58.00 (45.00,73.00)
>21	205	0.00 (0.00, 4.00)	1.00(0.00, 4.00)	0.00 (0.00, 3.00)	54.00(33.50, 72.00)
*Z*		−0.704	−1.373	−0.860	−1.577
*P*		0.482	0.170	0.390	0.115
Grade					
Freshman	363	1.00 (0.00, 4.00)	1.00 (0.00, 5.00)	0.00 (0.00, 4.00)	60.00 (47.00, 73.00)
Sophomore	221	0.00 (0.00, 4.00)	1.00 (0.00, 5.00)	0.00 (0.00, 2.00)	60.00 (42.50, 74.00)
Junior	217	0.00 (0.00, 4.00)	1.00 (0.00, 4.50)	0.00 (0.00, 3.00)	53.00 (38.50, 70.00)
Senior	298	1.00 (0.00, 4.00)	1.00 (0.00, 5.00)	0.00 (0.00, 2.00)	54.00 (38.50, 72.00)
Fifth year	21	3.00 (1.00, 5.50)	2.00 (0.00, 5.50)	0.00 (0.00, 7.00)	64.00 (49.00, 75.00)
*H*		9.858	1.331	8.689	11.192
*P*		0.043	0.856	0.069	0.024

Kruskal-Wallis H tests revealed overall significant differences in anxiety (*H* = 9.858, *p* = 0.043) and resilience (*H* = 11.192, *p* = 0.024) scores across grades. However, *post hoc* pairwise comparisons with Bonferroni correction showed no significant differences between specific grades (all Adj. *p* > 0.050). No statistically significant differences were observed across age or ethnicity groups (all *p* > 0.050).

### 3.3 Bullying exposure according to demographic characteristics

Among the 1,120 participants, 203 students reported traditional bullying victimization (18.1, 95% *CI* = 15.9–20.4%), and 62 acknowledged traditional bullying perpetration (5.5, 95% *CI* = 4.2–6.9%). Cyberbullying victimization was indicated by 133 students (11.9, 95% *CI* = 10.0–13.8%), while cyberbullying perpetration was disclosed by 77 students (6.9, 95% *CI* = 5.4–8.4%). Any forms of bullying victimization and perpetration were reported by 241 students (21.5, 95% *CI* = 19.1–23.9%) and 101 students (9.0, 95% *CI* = 7.3–10.7%), respectively.

As shown in Figure 1, male students had a higher prevalence of traditional bullying perpetration, cyberbullying perpetration, and total perpetration than female students (all *p* < 0.001), whereas no significant gender differences were observed in traditional bullying victimization, cyberbullying victimization, or total victimization (all *p* > 0.050). Traditional bullying perpetration was more prevalent among students aged >21 years than among those aged 18–21 years (*p* = 0.001). Significant grade differences were observed in traditional bullying victimization (*p* = 0.032), traditional bullying perpetration (*p* = 0.008), and total victimization (*p* = 0.021), but not in cyberbullying victimization, cyberbullying perpetration, or total perpetration (all *p* > 0.050). No significant differences by family residence or ethnicity were observed for any form of bullying exposure (all *p* > 0.050).

**Figure 1:**
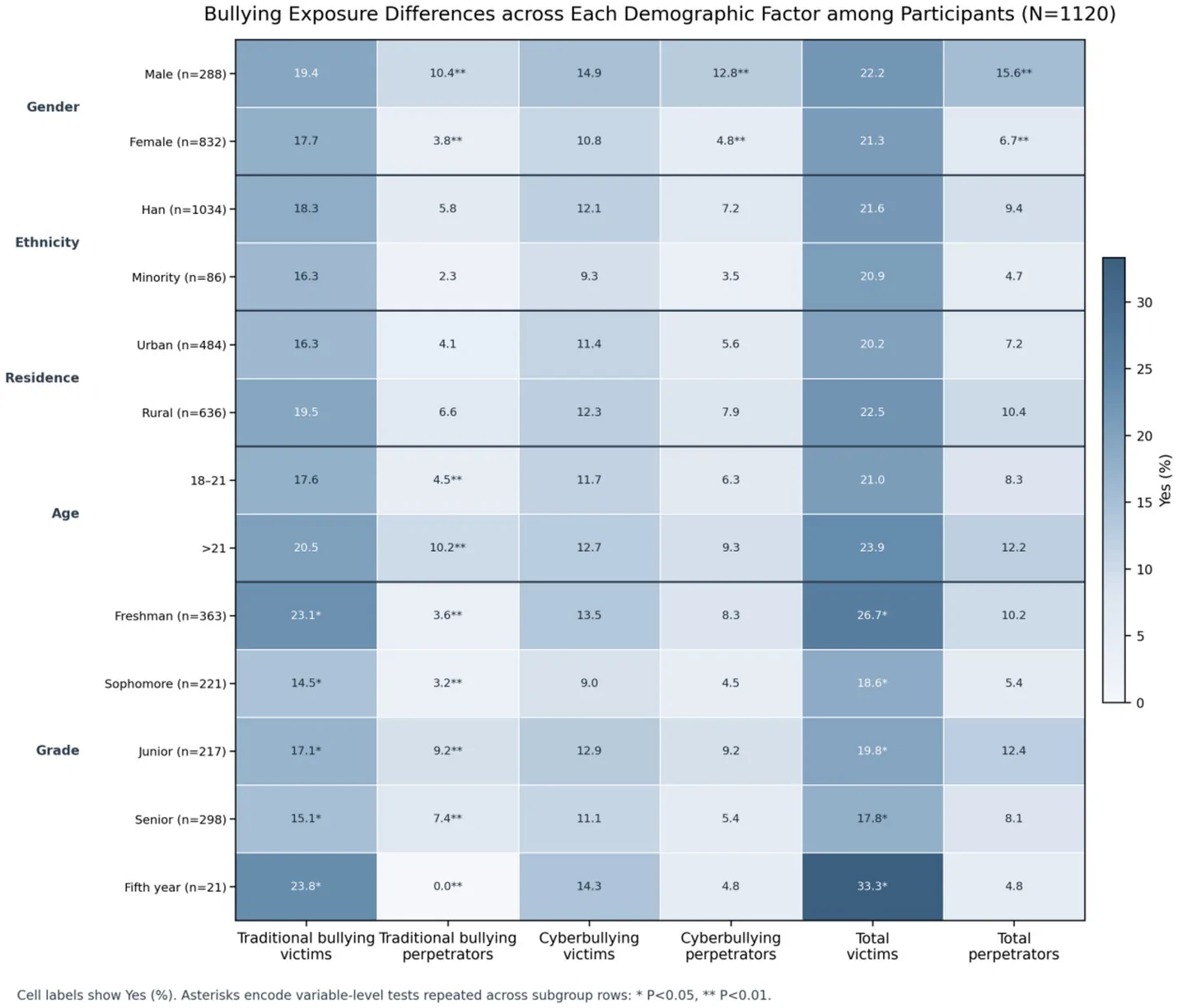
Bullying exposure by demographic characteristics among participants (*n* = 1,120).

### 3.4 Mental health according to bullying status

Participants were grouped separately according to each type of bullying exposure. Results showed (see Table 5) that students with traditional bullying victimization, traditional bullying perpetration, cyberbullying victimization, cyberbullying perpetration, total victimization, or total perpetration had significantly higher GAD-7, PHQ-9, and PCL-5 scores than those without the corresponding exposure, while their CD-RISC-25 scores were significantly lower. All differences were statistically significant (all *p* < 0.001).

**Table 5:** Mental health differences by bullying exposure among participants (n = 1,120).

Variable	N	Anxiety *Mdn* (*P_25_*, *P_75_*)	Depression *Mdn* (*P_25_*, *P_75_*)	PTSD *Mdn* (*P_25_*, *P_75_*)	Resilience *Mdn* (*P_25_*, *P_75_*)
Traditional bullying victimization					
Yes	203	4.00 (1.00, 7.00)	5.00 (1.00, 9.00)	4.00 (0.00, 18.00)	48.00 (32.00, 66.00)
No	917	0.00 (0.00, 3.00)	1.00 (0.00, 4.00)	0.00 (0.00, 1.00)	60.00 (46.00, 74.00)
*Z*		10.001	9.867	12.696	−5.416
*P*		<0.001	<0.001	<0.001	<0.001
Traditional bullying perpetration					
Yes	62	6.00 (1.75, 9.00)	6.00 (2.75, 9.50)	12.00 (2.00, 25.25)	40.00 (18.75, 59.25)
No	1,058	1.00 (0.00, 4.00)	1.00 (0.00, 4.00)	0.00 (0.00, 2.00)	58.00 (46.00, 73.00)
*Z*		6.399	6.767	9.080	−5.444
*P*		<0.001	<0.001	<0.001	<0.001
Cyberbullying victimization					
Yes	133	5.00 (1.00, 7.00)	6.00 (2.00, 9.00)	7.00 (1.00, 20.00)	48.00 (34.00, 67.50)
No	987	0.00 (0.00, 3.00)	1.00 (0.00, 4.00)	0.00 (0.00, 2.00)	58.00 (45.00, 73.00)
*Z*		8.778	9.579	11.769	−3.519
*P*		<0.001	<0.001	<0.001	<0.001
Cyberbullying perpetration					
Yes	77	6.00 (2.00, 9.00)	7.00 (3.00, 9.00)	9.00(1.00, 23.00)	49.00 (31.50, 64.50)
No	1,043	0.00 (0.00, 4.00)	1.00 (0.00, 4.00)	0.00 (0.00, 2.00)	58.00 (45.00, 73.00)
*Z*		7.679	8.472	8.781	−3.377
*P*		<0.001	<0.001	<0.001	<0.001
Total victimization					
Yes	241	4.00 (1.00, 7.00)	5.00 (1.00, 9.00)	4.00 (0.00, 18.00)	50.00 (35.00, 68.50)
No	879	0.00 (0.00, 3.00)	0.00 (0.00, 4.00)	0.00 (0.00, 1.00)	60.00(46.00, 74.00)
*Z*		10.365	10.720	12.963	−4.747
*P*		<0.001	<0.001	<0.001	<0.001
Total perpetration					
Yes	101	5.00 (1.00, 9.00)	6.00 (3.00, 9.00)	7.00 (1.50, 21.50)	47.00 (26.00, 62.00)
No	1,019	0.00 (0.00, 4.00)	1.00 (0.00, 4.00)	0.00 (0.00, 2.00)	58.00(46.00, 73.00)
*Z*		8.207	9.058	10.548	−5.113
*P*		<0.001	<0.001	<0.001	<0.001

### 3.5 Correlations between bullying exposure and mental health

As shown in Table 6, Spearman correlation analyses reveal that all categories of bullying involvement are significantly and positively associated with anxiety, depression, and PTSD, with correlation coefficients ranging from 0.195 to 0.392. In contrast, these variables exhibit significant negative associations with psychological resilience, with r values ranging from −0.169 to −0.103 (all *p* < 0.01). Furthermore, the analysis indicates positive correlations among the four bullying categories. Traditional perpetration is associated with cyber perpetration (*r* = 0.539) and traditional victimization (*r* = 0.467), while the correlation is observed between cyber perpetration and cyber victimization (*r* = 0.560). These results demonstrate a substantial overlap and behavioural co-occurrence across different bullying roles and platforms.

**Table 6:** Spearman correlation matrix of bullying exposure and mental health indicators among participants (*n* = 1,120).

Variables	1	2	3	4	5	6	7	8
1. Traditional bullying victimization	1	0.467**	0.543**	0.427**	0.309**	0.304**	0.392**	−0.169**
2. Traditional bullying perpetration		1	0.432**	0.539**	0.195**	0.205**	0.274**	−0.163**
3. Cyberbullying victimization			1	0.560**	0.269**	0.293**	0.359**	−0.108**
4. Cyberbullying perpetration				1	0.232**	0.256**	0.266**	−0.103**
5. Anxiety					1	0.786**	0.631**	−0.192**
6. Depression						1	0.666**	−0.201**
7. PTSD							1	−0.180**
8. Resilience								1

**p < 0.01.

### 3.6 Univariable regression analyses of bullying exposure associated with mental health

Model 1 was established via univariable regression analysis to explore factors associated with anxiety, depression, PTSD, and psychological resilience. In these analyses, the total scores of anxiety, depression, PTSD, and resilience were treated as dependent variables, while demographic characteristics, traditional bullying perpetration, traditional bullying victimization, cyberbullying perpetration, and cyberbullying victimization were included as independent variables. Results (see Table 7) showed that all four types of bullying behaviours were significantly associated with higher levels of anxiety, depression, and PTSD (all *p* < 0.050). With respect to resilience, these four bullying variables were significantly linked to lower resilience scores (*p* < 0.050). Among demographic characteristics, age was negatively associated with resilience (*B* = −4.129, 95% *CI* = [−8.102, −0.156], *p* = 0.042), and senior students had lower resilience scores than freshmen (*B* = −3.576, 95% *CI* = [−7.053, −0.099], *p* = 0.044). No other demographic variables were significantly associated with the four outcomes (all *p* > 0.050).

**Table 7:** Univariable regression analyses of variables associated with mental health indicators among participants (*n* = 1,120).

Variable		*B*	*SE*	*β*	*t*	*P*	95% *CI*	*R^2^*	*F*
Anxiety	Traditional bullying victimization	0.646	0.053	0.342	12.168	<0.001	[0.542, 0.750]	0.117	148.07
	Traditional bullying perpetration	0.590	0.067	0.255	8.823	<0.001	[0.459, 0.721]	0.065	77.843
	Cyberbullying victimization	0.526	0.046	0.326	11.517	<0.001	[0.436, 0.616]	0.106	132.634
	Cyberbullying perpetration	0.423	0.047	0.261	9.041	<0.001	[0.331, 0.514]	0.068	81.732
	Gender	0.324	0.250	0.039	1.294	0.196	[−0.167, 0.815]	0.001	1.674
	Ethnicity	−0.490	0.411	−0.036	−1.193	0.233	[−1.297, 0.316]	0.001	1.423
	Family residence	−0.408	0.221	−0.055	−1.848	0.065	[−0.841, 0.025]	0.003	3.416
	Age	−0.059	0.283	−0.006	−0.210	0.834	[−0.615, 0.496]	0.000	0.044
	Grade (ref freshman)								
	Sophomore	−0.063	0.275	−0.007	−0.227	0.820	[−0.603, 0.477]	0.000	0.052
	Junior	−0.313	0.277	−0.034	−1.131	0.258	[−0.857, 0.230]	0.001	1.279
	Senior	0.100	0.248	0.012	0.405	0.685	[−0.386, 0.587]	0.000	0.164
	Fifth year	1.457	0.806	0.054	1.807	0.071	[−0.125, 3.039]	0.003	3.264
Depression	Traditional bullying victimization	0.738	0.061	0.340	12.080	<0.001	[0.618, 0.858]	0.115	145.933
	Traditional bullying perpetration	0.661	0.077	0.248	8.577	<0.001	[0.510, 0.812]	0.062	73.562
	Cyberbullying victimization	0.629	0.052	0.339	12.036	<0.001	[0.527, 0.732]	0.115	144.864
	Cyberbullying perpetration	0.490	0.054	0.263	9.121	<0.001	[0.385, 0.595]	0.069	83.196
	Gender	0.190	0.288	0.020	0.661	0.509	[−0.375, 0.756]	0.000	0.436
	Ethnicity	−0.500	0.473	−0.032	−1.057	0.291	[−1.428, 0.428]	0.001	1.118
	Family residence	−0.389	0.254	−0.046	−1.532	0.126	[−0.888, 0.109]	0.002	2.348
	Age	−0.361	0.326	−0.033	−1.110	0.267	[−1.000, 0.278]	0.001	1.231
	Grade (ref freshman)								
	Sophomore	0.079	0.316	0.008	0.251	0.802	[−0.542, 0.700]	0.000	0.063
	Junior	−0.130	0.319	−0.012	−0.409	0.682	[−0.756, 0.495]	0.000	0.167
	Senior	−0.055	0.285	−0.006	−0.192	0.848	[−0.614, 0.504]	0.000	0.037
	Fifth year	−0.124	0.929	−0.004	−0.134	0.893	[−1.946, 1.698]	0.000	0.018
PTSD	Traditional bullying victimization	2.219	0.142	0.423	15.587	<0.001	[1.939, 2.498]	0.179	242.944
	Traditional bullying perpetration	1.964	0.183	0.306	10.733	<0.001	[1.605, 2.323]	0.093	115.204
	Cyberbullying victimization	1.897	0.122	0.423	15.598	<0.001	[1.659, 2.136]	0.179	243.312
	Cyberbullying perpetration	1.417	0.128	0.315	11.100	<0.001	[1.167, 1.668]	0.099	123.221
	Gender	0.050	0.696	0.002	0.072	0.943	[−1.316, 1.416]	0.000	0.005
	Ethnicity	0.359	1.143	0.009	0.314	0.754	[−1.884, 2.602]	0.000	0.099
	Family residence	−0.274	0.614	−0.013	−0.446	0.655	[−1.480, 0.931]	0.000	0.199
	Age	0.467	0.787	0.018	0.593	0.553	[−1.078, 2.011]	0.000	0.351
	Grade (ref freshman)								
	Sophomore	−0.201	0.765	−0.008	−0.263	0.793	[−1.702, 1.299]	0.000	0.069
	Junior	−0.073	0.770	−0.003	−0.094	0.925	[−1.584, 1.438]	0.000	0.009
	Senior	−0.663	0.688	−0.029	−0.963	0.336	[−2.014, 0.688]	0.001	0.928
	Fifth year	0.242	2.244	0.003	0.108	0.914	[−4.160, 4.645]	0.000	0.012
Resilience	Traditional bullying victimization	−1.875	0.401	−0.139	−4.677	<0.001	[−2.662, −1.088]	0.019	21.876
	Traditional bullying perpetration	−2.06	0.492	−0.124	−4.191	<0.001	[−3.025, −1.096]	0.015	17.562
	Cyberbullying victimization	−1.008	0.345	−0.087	−2.923	0.004	[−1.684, −0.331]	0.008	8.545
	Cyberbullying perpetration	−1.022	0.345	−0.088	−2.959	0.003	[−1.700, −0.345]	0.008	8.758
	Gender	2.472	1.793	0.041	1.378	0.168	[−1.047, 5.991]	0.002	1.900
	Ethnicity	−2.072	2.946	−0.021	−0.704	0.482	[−7.852, 3.708]	0.000	0.495
	Family residence	2.637	1.582	0.050	1.667	0.096	[−0.466, 5.741]	0.002	2.780
	Age	−4.129	2.025	−0.061	−2.039	0.042	[−8.102, −0.156]	0.004	4.157
	Grade (ref freshman)								
	Sophomore	0.898	1.971	0.014	0.455	0.649	[−2.970, 4.765]	0.000	0.207
	Junior	−3.651	1.982	−0.055	−1.842	0.066	[−7.539, 0.238]	0.003	3.393
	Senior	−3.576	1.772	−0.060	−2.018	0.044	[−7.053, −0.099]	0.004	4.072
	Fifth year	5.758	5.781	0.030	0.996	0.319	[−5.585, 17.101]	0.001	0.992

### 3.7 Multivariable regression analyses of bullying exposure and mental health

A multivariable linear regression model was then constructed, incorporating variables that demonstrated statistical significance in the univariable analysis (Model 1), to examine independent associated factors for each outcome. As shown in Table 8, traditional bullying victimization and cyberbullying victimization were positively associated with both anxiety and depression scores (all *p* < 0.001), whereas traditional bullying perpetration was negatively associated with anxiety (*p* = 0.026) and depression (*p* = 0.004). Cyberbullying perpetration was not significantly associated with anxiety (*p* = 0.112) or depression (*p* = 0.060). Regarding PTSD, traditional bullying victimization and cyberbullying victimization were positively associated with symptom scores, whereas traditional bullying perpetration and cyberbullying perpetration were negatively associated with symptom scores (all *p* < 0.001). Traditional bullying victimization was the only variable significantly associated with resilience, with greater victimization associated with lower resilience scores (*B* = −1.817, 95% *CI* = [−3.178, −0.456], *p* = 0.009). The remaining variables in the resilience model were not statistically significant (all *p* > 0.050).

**Table 8:** Multivariable regression analyses of bullying exposure and mental health among participants (*n* = 1,120).

Variable		*B*	*SE*	*β*	*t*	*P*	95% *CI*	*R^2^*	*F*
Anxiety	Constant	−0.938	0.863	—	−1.088	0.277	[−2.631, 0.754]	0.140	45.254
	Traditional bullying victimization	0.549	0.091	0.290	6.057	<0.001	[0.371, 0.726]		
	Traditional bullying perpetration	−0.329	0.148	−0.142	−2.223	0.026	[−0.619, −0.039]		
	Cyberbullying victimization	0.533	0.109	0.330	4.888	<0.001	[0.319, 0.747]		
	Cyberbullying perpetration	−0.191	0.120	−0.118	−1.591	0.112	[−0.427, 0.045]		
Depression	Constant	−1.859	0.986	—	−1.885	0.060	[−3.794, 0.076]	0.150	49.017
	Traditional bullying victimization	0.609	0.104	0.280	5.882	<0.001	[0.406, 0.812]		
	Traditional bullying perpetration	−0.482	0.169	−0.181	−2.851	0.004	[−0.814, −0.150]		
	Cyberbullying victimization	0.745	0.125	0.401	5.974	<0.001	[0.500, 0.989]		
	Cyberbullying perpetration	−0.258	0.137	−0.139	−1.879	0.060	[−0.528, 0.011]		
PTSD	Constant	−9.605	2.253	—	−4.262	<0.001	[−14.026, −5.183]	0.240	87.819
	Traditional bullying victimization	1.807	0.237	0.344	7.637	<0.001	[1.343, 2.272]		
	Traditional bullying perpetration	−1.310	0.386	−0.204	−3.393	<0.001	[−2.068, −0.553]		
	Cyberbullying victimization	2.516	0.285	0.561	8.834	<0.001	[1.957, 3.075]		
	Cyberbullying perpetration	−1.150	0.314	−0.256	−3.664	<0.001	[−1.766, −0.534]		
Resilience	Constant	46.859	6.635	—	7.062	<0.001	[33.840, 59.878]	0.026	5.023
	Traditional bullying victimization	−1.817	0.694	−0.134	−2.620	0.009	[−3.178, −0.456]		
	Traditional bullying perpetration	−1.566	1.137	−0.095	−1.377	0.169	[−3.796, 0.665]		
	Cyberbullying victimization	0.599	0.832	0.052	0.720	0.472	[−1.034, 2.231]		
	Cyberbullying perpetration	0.546	0.917	0.047	0.596	0.551	[−1.253, 2.346]		
	Age	−1.998	2.153	−0.029	−0.928	0.354	[−6.223, 2.227]		
	Grade (ref freshman)								
	Senior	−2.841	1.881	−0.048	−1.510	0.131	[−6.531, 0.850]		

### 3.8 Sensitivity analysis and collinearity diagnostics

Model 3 simultaneously incorporated all variables for sensitivity analysis (Table 9). Traditional bullying victimization and cyberbullying victimization were significantly associated with higher anxiety levels (all *p* < 0.001), while traditional bullying perpetration was significantly associated with lower anxiety scores. Gender, ethnicity and fifth-year grade status were also significantly associated with anxiety scores (all *p* < 0.050). For depression, traditional bullying victimization and cyberbullying victimization were significantly associated with higher depression scores, whereas traditional bullying perpetration showed a significant association with lower depression levels. Age was also significantly associated with anxiety and depression scores (all *p* < 0.050).

**Table 9:** Sensitivity analysis of bullying exposure and mental health among participants (*n* = 1,120).

Variable		*B*	*SE*	*β*	*t*	*P*	95% *CI*	*R^2^*	*F*
Anxiety	Constant	−0.760	1.078	—	−0.705	0.481	[−2.876, 1.356]	0.158	17.259
	Traditional bullying victimization	0.548	0.091	0.290	6.045	<0.001	[0.370, 0.725]		
	Traditional bullying perpetration	−0.298	0.149	−0.129	−2.005	0.045	[−0.589, −0.006]		
	Cyberbullying victimization	0.537	0.109	0.333	4.951	<0.001	[0.324, 0.750]		
	Cyberbullying perpetration	−0.183	0.120	−0.113	−1.529	0.127	[−0.418, 0.052]		
	Gender	0.521	0.234	0.062	2.225	0.026	[0.062, 0.981]		
	Ethnicity	−0.814	0.389	−0.059	−2.095	0.036	[−1.577, −0.051]		
	Family residence	−0.288	0.206	−0.039	−1.399	0.162	[−0.693, 0.116]		
	Age	−0.642	0.311	−0.068	−2.062	0.039	[−1.253, −0.031]		
	Grade (ref freshman)								
	Sophomore	0.027	0.290	0.003	0.094	0.925	[−0.542, 0.596]		
	Junior	−0.339	0.303	−0.037	−1.119	0.263	[−0.934, 0.255]		
	Senior	0.359	0.293	0.043	1.227	0.220	[−0.215, 0.933]		
	Fifth-year	1.857	0.806	0.069	2.302	0.021	[0.274, 3.439]		
Depression	Constant	−1.431	1.237	—	−1.156	0.248	[−3.859, 0.997]	0.161	17.699
	Traditional bullying victimization	0.617	0.104	0.284	5.937	<0.001	[0.413, 0.821]		
	Traditional bullying perpetration	−0.454	0.170	−0.171	−2.662	0.008	[−0.788, −0.119]		
	Cyberbullying victimization	0.745	0.125	0.401	5.982	<0.001	[0.501, 0.989]		
	Cyberbullying perpetration	−0.252	0.137	−0.135	−1.833	0.067	[−0.521, 0.018]		
	Gender	0.422	0.269	0.044	1.569	0.117	[−0.106, 0.949]		
	Ethnicity	−0.861	0.446	−0.054	−1.929	0.054	[−1.736, 0.015]		
	Family residence	−0.286	0.237	−0.032	−1.133	0.257	[−0.732, 0.196]		
	Age	−0.826	0.357	−0.076	−2.310	0.021	[−1.527, −0.124]		
	Grade (ref freshman)								
	Sophomore	0.154	0.333	0.015	0.462	0.644	[−0.499, 0.806]		
	Junior	−0.194	0.348	−0.018	−0.557	0.578	[−0.876, 0.489]		
	Senior	0.339	0.336	0.036	1.009	0.313	[−0.320, 0.998]		
	Fifth-year	0.433	0.925	0.014	0.468	0.640	[−1.383, 2.249]		
PTSD	Constant	−10.474	2.840	—	−3.687	<0.001	[−16.047, −4.901]	0.243	29.607
	Traditional bullying victimization	1.794	0.239	0.342	7.522	<0.001	[1.326, 2.263]		
	Traditional bullying perpetration	−1.252	0.391	−0.195	−3.200	0.001	[−2.020, −0.484]		
	Cyberbullying victimization	2.520	0.286	0.562	8.818	<0.001	[1.960, 3.081]		
	Cyberbullying perpetration	−1.146	0.315	−0.255	−3.637	<0.001	[−1.764, −0.528]		
	Gender	0.872	0.617	0.037	1.413	0.158	[−0.339, 2.082]		
	Ethnicity	−0.412	1.024	−0.011	−0.403	0.687	[−2.422, 1.597]		
	Family residence	0.025	0.543	0.001	0.046	0.963	[−1.041, 1.091]		
	Age	0.190	0.820	0.007	0.231	0.817	[−1.420, 1.799]		
	Grade (ref freshman)								
	Sophomore	−0.082	0.764	−0.003	−0.108	0.914	[−1.581, 1.416]		
	Junior	−1.224	0.798	−0.048	−1.534	0.125	[−2.790, 0.342]		
	Senior	−0.601	0.771	−0.026	−0.779	0.436	[−2.113, 0.912]		
	Fifth-year	−0.783	2.124	−0.010	−0.368	0.713	[−4.950, 3.385]		
Resilience	Constant	44.732	8.265	—	5.412	<0.001	[28.516, 60.948]	0.035	3.380
	Traditional bullying victimization	−1.908	0.694	−0.141	−2.748	0.006	[−3.270, −0.546]		
	Traditional bullying perpetration	−1.354	1.138	−0.082	−1.190	0.234	[−3.588, 0.879]		
	Cyberbullying victimization	0.599	0.832	0.052	0.720	0.472	[−1.033, 2.231]		
	Cyberbullying perpetration	0.608	0.917	0.052	0.664	0.507	[−1.190, 2.407]		
	Gender	1.574	1.795	0.026	0.877	0.381	[−1.948, 5.096]		
	Ethnicity	−2.898	2.980	−0.029	−0.972	0.331	[−8.746, 2.950]		
	Family residence	2.380	1.580	0.045	1.506	0.132	[−0.721, 5.481]		
	Age	−1.547	2.387	−0.023	−0.648	0.517	[−6.231, 3.137]		
	Grade (ref freshman)								
	Sophomore	−2.668	2.222	−0.040	−1.201	0.230	[−7.028, 1.692]		
	Junior	−5.233	2.322	−0.079	−2.254	0.024	[−9.789, −0.677]		
	Senior	−4.935	2.243	−0.083	−2.200	0.028	[−9.336, −0.533]		
	Fifth-year	4.305	6.181	0.022	0.697	0.486	[−7.822, 16.432]		

Traditional and cyberbullying victimization were significantly associated with higher PTSD symptom severity, while both traditional and cyberbullying perpetration showed significant associations with lower levels of these symptoms (all *p* < 0.001). Regarding resilience, traditional bullying victimization was significantly associated with lower resilience scores (*p* < 0.050). In addition, junior and senior students had significantly lower resilience scores than freshmen (all *p* < 0.050).

To ensure the reliability of the regression estimates, multicollinearity was assessed using the VIF. Cyberbullying perpetration, cyberbullying victimization, and traditional bullying perpetration exhibited higher VIF values compared to other covariates, reflecting the substantial overlap and co-occurrence between different bullying roles and across platforms. Despite these elevated values, all VIF results remained <10 (58), indicating that the multivariable models were not substantially biased by multicollinearity. The negative associations observed for traditional bullying perpetration across anxiety, depression, and PTSD, as well as for cyberbullying perpetration within the PTSD model, represent independent associations after statistically adjusting for all other variables. This suggests that due to the high correlation between roles, once the variance associated with victimization is accounted for in the model, perpetration behaviors exhibit negative associations with these mental health outcomes.

## 4 Discussion

Given the high prevalence of bullying victimization and its strong associations with anxiety, depression, PTSD, and reduced psychological resilience, these findings underscore the need for universities to develop categorized, tiered, and targeted mental health interventions. Several factors may account for these variations, including methodological approaches, sample characteristics, regional contexts, and temporal differences in data collection. These contextual considerations are essential for interpreting the present findings in relation to the existing literature. The bullying victimization rate in this study (21.5%) falls within the 8.09–39.2% range reported in previous Chinese research and is lower than the multi-university survey. A multi-university survey in Hefei, Anhui province found that 59.7% of college students had experienced bullying exposure during their lifetime (33). More recently, a study of Chinese undergraduates reported a past-year prevalence of 27.4%, including 15.6% for traditional bullying and 15.2% for cyberbullying (14). Guo (59) reported a cyberbullying victimization prevalence of 26.45% among students from ten colleges in Liaoning province. Internationally, a study among medical and health sciences university students in the United Arab Emirates reported a bullying victimization prevalence of 34.1% (60), whereas a Swedish national study across 38 higher education institutions reported a bullying victimization prevalence of 5.43% (61). This discrepancy may be explained by several factors. First, regional differences are noteworthy: our study was conducted in Sichuan Province, where local cultural norms, educational environments, and socioeconomic conditions may differ from other regions. Second, variations in measurement instruments and operational definitions of bullying may influence prevalence estimates. Third, temporal factors are relevant: data were collected in 2025, during a period characterized by increased digital engagement and social media usage, which may have expanded both bullying opportunities and awareness. Additionally, our sample exclusively comprised students from a single medical college, which may experience unique competitive academic pressures and stress levels compared to comprehensive universities, potentially contributing to higher reported rates of bullying involvement.

A further pattern that warrants interpretation is why victimization showed stronger and more consistent associations with anxiety and depression than perpetration. Three complementary explanations are plausible. First, from the perspective of stress-appraisal theory, victimization constitutes a direct interpersonal threat directed at the self. It activates primary appraisals of threat and harm that elicit fear, hypervigilance, and anticipatory worry and secondary appraisals of insufficient coping resources that engender helplessness, self-blame, and negative internalized attributions characteristic of depression. These inward-directed (internalizing) responses map closely onto the constructs measured by the GAD-7 and PHQ-9. Second, perpetration reflects an outward-directed (externalizing) pattern; although perpetration carries its own developmental risks, including conduct problems, reduced empathy, and later antisocial outcomes, it is less directly tied to internalizing distress, and the perpetrator does not experience the same threat-to-self that the victim does. Third, part of the discrepancy is methodological: because perpetration and victimization are substantially correlated, reflecting the bully–victim overlap, the perpetration coefficient is attenuated—and can even reverse sign—once victimization is statistically controlled, as elaborated below. Collectively, these considerations explain why victimization, rather than perpetration, emerged as the more robust correlate of anxiety and depression in the multivariable models.

In the correlation analyses and univariable regression analyses, all four bullying variables were associated with higher anxiety, depression, and PTSD scores and lower psychological resilience scores. After adjustment for demographic characteristics and overlapping bullying involvement, the associations involving victimization remained comparatively consistent, whereas the coefficients for perpetration were less stable and should be interpreted cautiously. The consistency of these associations across the primary and sensitivity models suggests that victimization was a comparatively robust correlate of psychological distress in the present sample. Previous studies have proposed that difficulties in emotion regulation, rumination, avoidant coping, and reduced perceived control may help explain the relationship between bullying victimization and poorer psychological functioning (38). However, these processes were not assessed in the present study and should therefore be regarded as possible explanatory pathways rather than mechanisms demonstrated by the current findings.

With respect to resilience levels, traditional bullying victimization was the only bullying-related variable significantly associated with lower psychological resilience in the multivariable analysis. Previous studies have suggested that resilience is not a completely fixed individual trait, but rather a dynamic psychological characteristic whose level may be related to stress experiences, interpersonal relationships, and social support (62, 63). Traditional bullying often occurs in face-to-face contexts, where victimized students may be more directly exposed to interpersonal stressors such as peer rejection, verbal aggression, and relational conflict (24). From a cognitive processing perspective, Wang et al. (64) found that negative cognitive processing bias mediated the association between bullying victimization and general psychological adaptation indicators. This finding indicates that negative cognitive processes can help us comprehend the link between bullying victimization and diminished psychological resilience (64). Conservation of Resources (COR) theory (52) provides a theoretical framework for understanding this association. This theory posits that individuals strive to acquire, maintain, and protect their psychological resources, and when these resources are depleted, stress and negative psychological outcomes ensue. Bullying victimization constitutes a chronic stressor that demands sustained mobilization of cognitive, emotional, and social resources to cope with harassment, intimidation, and social exclusion. As individuals repeatedly expend these resources without adequate opportunity for recovery, their resource reservoir gradually diminishes. This resource depletion compromises their capacity for adaptive coping and psychological recovery, ultimately manifested as reduced resilience scores. The theory conceptualizes resilience as a dynamic resource that can be eroded through prolonged exposure to adverse experiences such as bullying.

The multivariable models revealed negative associations between some perpetration-related variables and psychological symptom scores (e.g., traditional bullying perpetration with anxiety, depression, and PTSD, and cyberbullying perpetration with PTSD), and the associations involving cyberbullying perpetration were attenuated after adjustment for other bullying variables. These patterns must not be interpreted as evidence that bullying perpetration is protective or beneficial. Rather, they constitute a statistical suppression effect. Suppression arises when a predictor (perpetration) shares variance with another predictor (victimization) that is more strongly associated with the outcome; when this shared variance is partialled out, the residualized coefficient of the first predictor can be reduced, reversed in sign, or destabilized (65). In the present data, victimization and perpetration were substantially correlated, reflecting the well-documented overlap in which a considerable proportion of students both experience and enact bullying (66). Once victimization was entered into the model, it absorbed the shared distress-related variance, leaving perpetration with only its unique residual component. Because the residual “pure-perpetration” component (bullying others without being victimized) is comparatively weakly associated with internalizing distress, the perpetration coefficient reversed to a negative value. The negative coefficients should therefore be interpreted strictly as conditional estimates of perpetration after removing the distress variance it shares with victimization—not as causal or protective effects. Consistent with this interpretation, the zero-order correlations and univariable regressions showed that all four bullying variables, including perpetration, were positively associated with anxiety, depression, and PTSD.

These patterns should not be interpreted as evidence linking bullying perpetration to lower psychological risk. Rather, they may partly reflect overlap among bullying roles and forms. Previous studies have shown that victimization and perpetration roles, as well as traditional bullying and cyberbullying involvement, may co-occur within the same individuals; similarly, the observed correlation between traditional bullying perpetration and cyberbullying perpetration in the present study suggests shared behavioral variance, consistent with previous evidence that cyberbullying may overlap with traditional aggression across contexts (66). When correlated bullying variables are entered into the same multivariable model, shared variance may attenuate or destabilise specific regression coefficients, producing a possible suppression-like pattern (65). Therefore, perpetration-related coefficients should be interpreted as conditional estimates adjusted for overlapping bullying involvement. Although the VIF values did not indicate severe multicollinearity, VIF thresholds should not be interpreted mechanically, and meaningful predictor overlap may still affect coefficient estimates (67).

Sensitivity analysis incorporating all covariates provided additional support for the robustness of the primary findings. Traditional and cyberbullying victimization remained associated with higher anxiety, depression, and PTSD symptoms, while traditional bullying victimization remained associated with lower psychological resilience. These findings support the stability of the victimization-related results in the present sample.

College students are at a critical stage of personality development and social adaptation. Their mental health is closely related to academic functioning, social adaptation, and campus well-being (68). These findings may inform more differentiated and targeted approaches to campus mental health support. For victims of cyberbullying and elevated PTSD symptoms, timely psychological assessment, referral, and trauma-informed support may be considered. For victims of traditional bullying, emphasis may focus on enhancing psychological resilience through group counseling and training in social skills. In addition, students from rural backgrounds may need greater attention in campus adaptation support and mental health services, given their higher anxiety and depression scores and lower psychological resilience in this study. Thus, interventions for campus bullying may need to move beyond reactive handling and punishment toward risk identification and systemic prevention. Multidimensional strategies may help protect the mental health of college students, promote their healthy development, and enhance social adaptation.

### 4.1 Implications for practice and policy

The present findings carry several implications for institutional practice and policy. First, universities could incorporate bullying screening into routine mental health assessment. Brief, validated bullying-exposure and mental-health screening items could be embedded into routine student assessments, for example, at freshman orientation, annual physical and psychological check-ups, and counselling-centre intake. Given the strong associations between victimization and anxiety, depression, and PTSD observed here, systematic screening would facilitate early identification of both bullying involvement and co-occurring psychological distress. Screening should assess traditional and cyber forms and both victimization and perpetration roles, because bully–victims constitute a particularly high-risk subgroup; students screening positive should be triaged through a stepped-care pathway to appropriate counselling or clinical services.

Second, resilience-building interventions may help mitigate the adverse psychological consequences of bullying, particularly for victims of traditional bullying, which was the only bullying variable uniquely associated with lower psychological resilience. Consistent with Conservation of Resources theory, which conceptualizes resilience as a dynamic resource that chronic stress can deplete, interventions that replenish psychological resources and strengthen adaptive coping, including cognitive-behavioral skills training, mindfulness-based stress reduction, social-skills and assertiveness training, and group counselling, may attenuate the psychological impact of victimization.

Third, university policy should address traditional and cyberbullying through a single, integrated framework rather than treating them as separate problems, given their substantial co-occurrence in this sample. This includes a clear, publicized anti-bullying policy with safe and confidential reporting channels; bystander-intervention training; digital-citizenship and cyberbullying-prevention education; and trauma-informed support services, particularly for cyberbullying victims, who showed the strongest association with PTSD symptoms (69). Institutional measures such as training faculty and residential staff to recognize and respond to bullying, and routine monitoring of campus climate, would further support prevention. Finally, given that rural-origin students reported higher anxiety and depression and lower resilience, universities should strengthen adaptation and mental-health support for this subgroup. Collectively, these strategies would move campus responses beyond reactive handling and punishment toward risk identification, early intervention, and systemic prevention.

## 5 Strengths and limitations

This study addresses the limitations of previous broad categorizations of bullying types by distinguishing four dimensions of perpetration and victimization across both traditional and cyberbullying contexts. It simultaneously examines multidimensional indicators of anxiety, depression, PTSD, and psychological resilience, thereby elucidating the specific effects of various bullying types on mental health.

Several limitations of this study should be acknowledged. First, the cross-sectional design limits causal inference. Because bullying exposure and mental health outcomes were assessed concurrently, we cannot establish the temporal ordering of these associations, and the observed relationships may be bidirectional or influenced by unmeasured third variables. It is possible, for instance, that pre-existing anxiety, depression, or PTSD symptoms increase a student’s vulnerability to victimization as well as resulting from it. Longitudinal designs are therefore required to determine causal pathways and the direction of effects. Second, all measures relied on self-report, which is susceptible to recall bias and social desirability bias. These biases may lead to systematic underestimation of the prevalence of both perpetration and victimization and could attenuate or inflate the observed associations; future studies could incorporate multi-informant reports and objective or behavioral indicators of cyberbullying to triangulate self-report data. Third, the sample was drawn from a single medical college in Sichuan Province using convenience sampling, which limits the representativeness of the findings for the broader population of Chinese college students; medical students may experience distinct academic pressures, and the regional socio-cultural context may differ from other provinces. Fourth, the overrepresentation of female participants (74.3%), although reflective of the gender composition of the recruited institution, limits generalizability to male students, and the relatively small minority-ethnicity subgroup (7.7%) limits subgroup analyses. Fifth, although we adjusted for demographic covariates, potential confounding variables may not have been fully accounted for; unmeasured factors such as personality traits (e.g., neuroticism), social support, family functioning, prior mental health history, academic stress, sleep quality, substance use, and childhood adversity could confound the associations between bullying and mental health outcomes, and this residual confounding should be considered when interpreting the findings. Sixth, the study cannot determine the temporal relationship between bullying and mental health outcomes, a fundamental consequence of the cross-sectional design noted above. Finally, the high intercorrelations among the four bullying variables, although not exceeding conventional VIF thresholds, introduced a suppression pattern that complicates the interpretation of perpetration-specific coefficients. Multi-center studies with probability-based sampling across diverse regions and institution types are needed to establish the national generalizability of these findings.

Several directions for future research emerge from the present findings. First, longitudinal studies with repeated measures, including cross-lagged panel and latent growth models, are needed to determine causal pathways between bullying experiences and mental health outcomes, to establish temporal precedence, and to test bidirectional (transactional) models in which distress predicts subsequent victimization as well as the reverse. Second, future research should extend the outcome space beyond internalizing symptoms to examine how bullying experiences influence academic performance, social adjustment, and longer-term career development among college students. Third, studies should identify protective factors that moderate the relationship between bullying victimization and psychological distress—such as psychological resilience, perceived social support, adaptive coping style, self-efficacy, parental and peer support, and institutional climate—to clarify for whom bullying is most harmful and to inform targeted intervention design. Fourth, randomized controlled trials and rigorous quasi-experimental studies are needed to evaluate the effectiveness of university-based anti-bullying and resilience-enhancement interventions in improving mental health outcomes, and to identify the active ingredients, optimal intensity, and delivery modalities of such programmes.

## Data Availability

The raw data supporting the conclusions of this article will be made available by the authors, without undue reservation.
